# Renal failure due to primary amyloidosis: a case report and literature review

**DOI:** 10.1590/S1516-31802011000300009

**Published:** 2011-05-05

**Authors:** Ramon Andrade Bezerra de Mello, Dania Sofia Neiva Marques Santos, Margarida Paula Rebelo Nunes Freitas-Silva, Joaquim Aguiar Andrade

**Affiliations:** I MD. Doctoral student of Medicine and Molecular Oncology, School of Medicine, University of Porto, and Resident of Medical Oncology, “Francisco Gentil” Portuguese Institute of Oncology, Porto, Portugal.; II MD. Master's degree student of Clinical Oncology, Abel Salazar Institute of Biomedical Sciences, University of Porto, Porto, Portugal, in association with Thomas Jefferson University, United States.; III MD, MSc. Assistant professor, Department of Medicine, School of Medicine, University of Porto, and Specialist in Internal Medicine, Hospital São João, Porto, Portugal.; IV MD. Specialist in Clinical Hematology, Hospital São João, Porto, Portugal.

**Keywords:** Amyloidosis, Amyloid, Kidney failure, Nephrotic syndrome, Congo red, Amiloidose, Amilóide, Insuficiência renal, Síndrome nefrótica, Vermelho congo

## Abstract

**CONTEXT:**

Primary amyloidosis, also known as AL amyloidosis, is commonly caused by clonal expansion of plasma cells in the bone marrow, thereby segregating light chains of clonal immunoglobulin that settle in tissues in the form of insoluble amyloid fibrils. The aim of this study was to report a case of primary amyloidosis with renal failure, diagnosed in Hospital São João, Porto, Portugal, focusing on the diagnostic difficulties and presenting a literature review.

**CASE REPORT:**

A 68-year-old Caucasian man was admitted to the Internal Medicine Department of the hospital with a condition of anasarca and nephrotic syndrome. After performing a renal biopsy that tested positive using Congo red and immunohistochemistry, lambda light chain amyloidosis was diagnosed. This evolved into terminal renal disease, which led to hemodialysis and several episodes of urinary and catheter infections. He was started on chemotherapy, consisting of bortezomib 0.7 mg/m^2^ and dexamethasone 40 mg in six cycles. This led to clinical improvement, stabilization of the illness and good tolerance of the treatment.

**CONCLUSION:**

Amyloidosis is a rare entity that is difficult to diagnose. This is because of the unspecific early clinical manifestations of the disease. The hypothesis of amyloidosis is only considered when specific organ failure occurs. This case consisted of primary amyloidosis with involvement of the kidneys as an initial presentation of the disease and its difficulties were shown, going from the clinical approach to the final diagnosis.

## RESUMO

**CONTEXTO:**

A amiloidose primária, também conhecida como amiloidose AL, é geralmente causada pela expansão clonal de plasmócitos na medula óssea que segregam cadeias leves de imunoglobulina clonal, as quais se depositam nos tecidos na forma de fibrilas amiloides insolúveis. O objetivo deste estudo é relatar um caso de amiloidose primária com acometimento renal diagnosticado no Hospital São João, Porto, Portugal, enfatizando as dificuldades do diagnóstico e apresentando uma revisão da literatura.

**RELATO DO CASO:**

Homem de 68 anos, branco, foi admitido no Serviço de Medicina Interna do hospital com quadro de anasarca e síndrome nefrótica. Após realizar biópsia renal, que foi positiva para o vermelho congo e imunoistoquímica, foi diagnosticada amiloidose de cadeia leve lambda. Evoluiu para doença renal terminal, o que levou a hemodiálise e tendo vários episódios de infecções urinárias e do cateter. Iniciou a quimioterapia com bortezimib, 0,7 mg/m^2^, e dexametasona, 40 mg em seis ciclos, levando a uma melhoria clínica, a estabilização da doença e boa tolerância ao tratamento.

**CONCLUSÃO:**

Amiloidose consiste em uma entidade rara e de difícil diagnóstico. Isso ocorre devido a manifestações clínicas da doença pouco específicas, e esta hipótese só é considerada quando do acometimento de um órgão em particular. O caso em questão refere-se a uma apresentação da amiloidose primária com envolvimento renal, como apresentação clínica inicial da doença, e as dificuldades desde a abordagem clínica até o diagnóstico final.

## INTRODUCTION

Primary amyloidosis, also known as amyloid light (AL) chain disease, is most commonly caused by clonal expansion of plasma cells in the bone marrow, thereby segregating light chains of clonal immunoglobulin that settle in tissues in the form of insoluble amyloid fibrils.^1^ The progressive accumulation of amyloid deposits in normal tissues results in structural dysfunction, evolving into failure of the affected organ, most commonly the kidney, heart, liver and peripheral nervous system. It may be associated with multiple myeloma or other B-cell lymphoproliferative diseases, like non-Hodgkin lymphoma, Waldenstrom hypergammaglobulinemia or monoclonal gammopathy of undetermined significance (MGUS).^1,2^ If untreated, AL amyloidosis has a mortality rate of approximately 80% over a two-year period. Meanwhile, treatments that suppress monoclonal light chain immunoglobulin frequently result in a clinical improvement, with stabilization or regression of the amyloid deposits, thus resulting in improvement and preservation of multiorganic function.^2^ The new therapies are quite promising and have contributed greatly towards improvement of patients’ quality of life and prognosis. In the United States, a study showed that the estimated incidence was around 5.1 to 12.8 per million inhabitants per year. In another study conducted on 474 patients at the Mayo Clinic,^3^ it was estimated that 60% of the cases occurred over the age range from 50 to 70 years, and only 10% at ages below 50 years.^4^ This diagnosis occurs in about 2.5% of all native renal biopsies and it is the cause of death of 1 in every 1,500 people in the United Kingdom.^2,4^ It is therefore an extremely rare pathological condition and is often misdiagnosed. In some parts of the world, it leads to high morbidity and mortality rates when not treated adequately. The aim of this study was to report a case of primary amyloidosis with renal failure that was diagnosed in Hospital São João, Porto, Portugal, focusing on the diagnostic difficulties and presenting a literature review.

## CASE REPORT

A 68-year-old Caucasian man living in Porto, who had previously been autonomous regarding his daily activities, was admitted to the Emergency Department of Hospital São João on August 4, 2009, presenting complaints of two months of severe dyspnea after minimal effort, with anasarca, progressive asthenia and mild atypical thoracic pain in the left hemithorax. He had a previous medical history of chronic gastritis, systemic arterial hypertension (which had been diagnosed 15 years earlier and had been medicated and controlled), a diffuse degenerative osteomuscular pathological condition (with sporadic used of non-steroid anti-inflammatory drugs), sleep apnea corrected with surgery and benign prostatic hyperplasia. He was medicated with lisinopril in association with hydrochlorothiazide, lorazepam, finasteride, lansoprazole and furosemide. While under observation, he seemed to be aware, well-oriented and cooperative in time and space, and was hemodynamically stable. Pulmonary auscultation reveled diminished bilateral breathing sounds, associated with diffuse crackles (which were more accentuated in the expiratory phase), and also anasarca. An initial analytical assessment was made, which revealed renal failure and nephrotic proteinuria (Table 1). An electrocardiogram revealed poor progression of R waves. A chest roentgenogram presented a small bilateral pleural effusion.

**Table 1:** Laboratory analysis carried out in August 2009

Laboratory data	Results	Reference values
Hemoglobin	16.4 g/dl	12 - 16
Hematocrit	46.2%	37 - 49
Leukocytes	6.86 × 10^9^/l	4.0 - 11.0
Platelets	215 × 10^9^/l	180 - 500
Blood gas analysis	pH = 7.514	7.35 - 7.45
	pO_2_ = 81.8 mmHg	> 70
	pCO_2_ = 32.8 mmHg	35 - 45
	HCO_3_^-^ = 25.8 mmol/l	22 - 26
Serum albumin	16.6 g/l	38 - 51
Serum total proteins	47 g/l	64 - 83
Urea	55 mg/dl	10 - 50
Creatinine	2.03 mg/dl	0.6 - 1.0
Brain natriuretic peptide	118 pg/ml	< 100
24 h proteinuria	9.48 g/l	< 0.15
Serum lambda integral chains	51.1 mg/dl	110 - 240
Serum kappa/lambda ratio	1.56	1.35 - 2.65
Urine lambda chains	6.3 mg/dl	Undetectable
Urine kappa/lambda ratio	1.06	0.75 - 4.50
Serum immunofixation	Apparent absence of monoclonal gammopathy	Undetectable
Urine immunofixation	Presence of free lambda light chains	Undetectable

**Figure 1:**
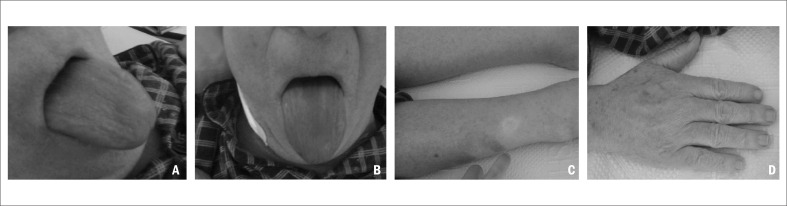
Clinical features: A and B show macroglossia; C shows the initial edemas; and D shows the upper extremities.

**Figure 2:**
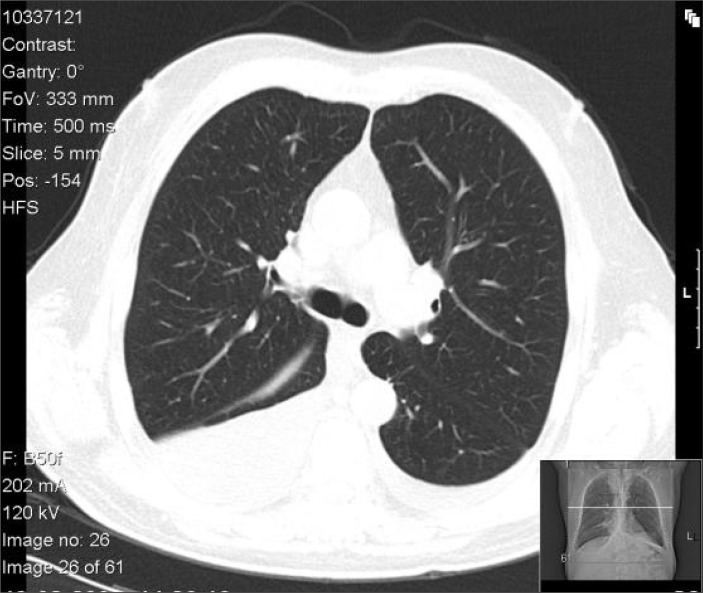
Features of computed tomography of the chest.

The patient was admitted to the Internal Medicine Department to evaluate the etiology of the nephrotic syndrome, among which the possibilities were: lupus nephritis, amyloidosis, nephropathy associated with human immunodeficiency virus (HIV), multiple myeloma or idiopathic origin. In the Internal Medicine ward, the patient still showed anasarca and now presented macroglossia (Figure 1). He was given diuretic therapy, without much response. Evaluations on auto-antibodies and HIV serology were negative. He presented normal hepatic function and coagulation. A thoracic computed tomography (CT) scan was performed (Figure 2) to screen for adjacent neoplasia, and this revealed an aerial bronchogram with condensation of the middle segment of the medial lobe. For this reason, he underwent bronchoscopy with bronchoalveolar lavage, which revealed *Enterobacter gergoviae* that was sensitive to sulfamethoxazole/trimethoprim. He was on this antibiotic for 10 days.

**Figure 3:**
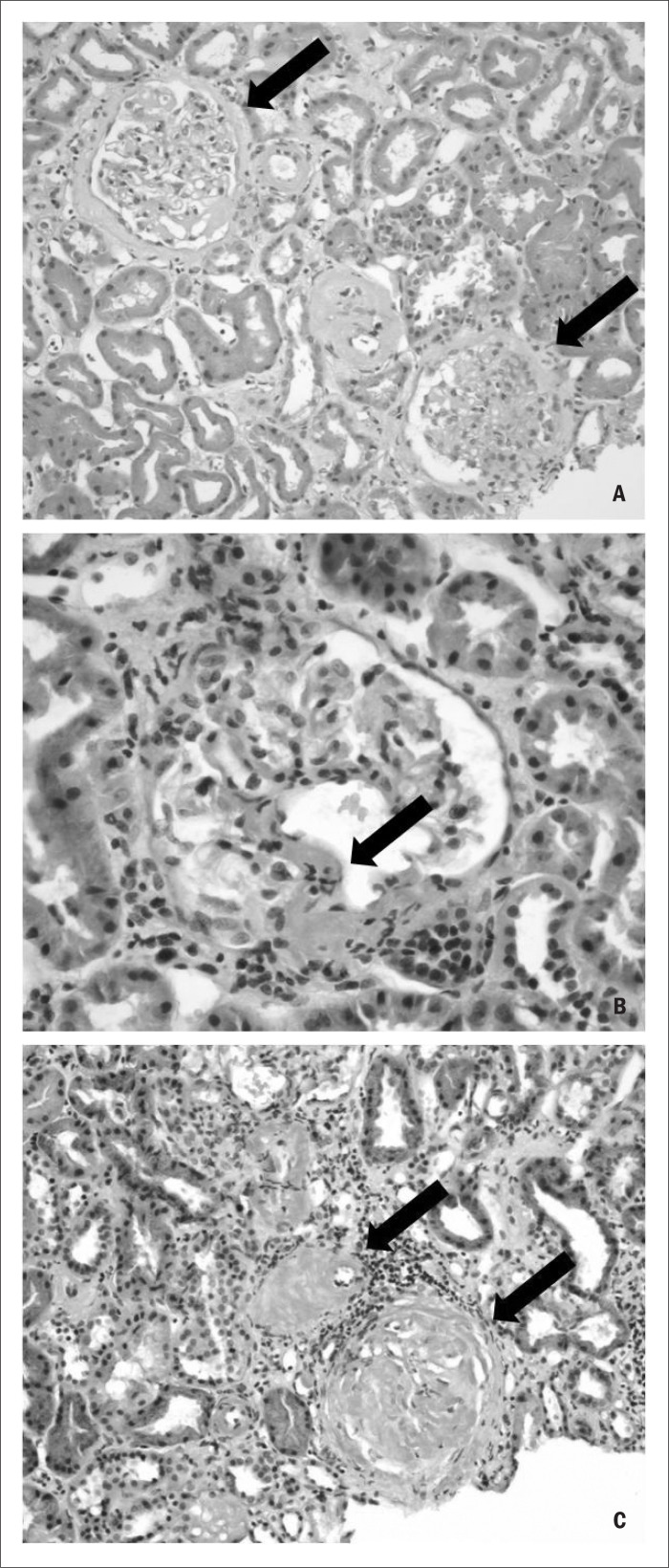
A) Hematoxylin and eosin stain showing the kidney cortex with glomerulus (arrows), microtubules and arterioles; B) showing the detail of Congo red stain (arrow); C) showing Congo red stain (arrows) marking amyloidal substance in renal mesangial matrix.

To screen for multiple myeloma, light chain and 24-hour urine immunofixation were performed, and this revealed free lambda light chains. A renal biopsy performed on August 7, 2009, revealed an image compatible with positive Congo red findings. Subsequently, under a polarizing optical microscope, apple-green birefringence was identified (Figure 3), thereby diagnosing renal amyloidosis. The patient's status improved and he was discharged with reinforcement of the diuretic therapy.

However, on September 1, 2009, the patient was admitted to the hospital again with an episode of kidney failure. An abdominal CT scan was performed to screen for another associated neoplasia, which turned out to be negative. He improved, and was discharged on September 15, 2009. Subsequent to this, he was again admitted, this time in the intensive care unit, with anasarca, severe renal failure and orthostatic hypotension. He started hemodialysis on October 9, 2009, by means of a central venous line. His status evolved to urine and catheter infections, which were treated with adequate antibiotic therapy, with subsequent resolution of the inflammatory markers.

In the second week of October, the diagnosis of lambda light chain amyloidosis was confirmed through immunohistochemistry. The patient was classified as a high-risk case because he presented stage V renal failure and high basal brain natriuretic peptide at an age older than 65 years. He was started on chemotherapy with six cycles of bortezomib 0.7 mg/m^2^ and dexamethasone 40 mg, and his condition improved, with good tolerance to the medication. After beginning the treatment, he also presented level IV+ hemiparesis in the left upper arm, which improved after rehabilitation treatment and over the course of the chemotherapy cycles. Currently, the patient is stable and the disease has stagnated.

## DISCUSSION

Amyloidosis is an extremely rare entity that is difficult to diagnose. This is because of the unspecific early clinical manifestations of the disease. The hypothesis of amyloidosis is only considered when specific organ failure occurs. The average age at diagnosis is 64 years, and this condition occurs mainly in men. One of the first clinical manifestations of amyloidosis is macroglossia, which only occurs in approximately 10% of all cases. It is considered to be a pathognomonic sign of the disease. However, other causes of macroglossia, like tongue cancer, hypothyroidism or vitamin B12 or folic acid deficiency also need to be investigated.^2,4,5^

Amyloidosis may be systemic or localized. The most common ways in which primary amyloidosis is presented are through nephrotic syndrome (which was initially observed in this case), peripheral neuropathy (which was also observed in this case), congestive cardiomyopathy and hepatomegaly. Fatigue and weight loss are very common, but usually they only occur when an organ is involved. This patient had complaints compatible with nephrotic syndrome over the two months prior to his first visit to the Emergency Department. Some patients also present conditions related to multiple myeloma, which was not the case here. This association has a poor prognosis and therapy is difficult. Renal and heart failure are the major causes of death.^2,5,6^ Almost every patient with AL amyloidosis has clonal B-cell dyscrasia, but monoclonal protein in the serum or urine is only identified in 85% of the cases.^4^

Once a diagnosis of amyloidosis is suspected, it should be confirmed through biopsy on the affected organ, with histopathological assessment using the Congo red technique. This technique was introduced by Bennhold in 1922; the amyloid substance has a red/brown coloration when observed in daylight, but the diagnosis is confirmed by the apple-green birefringence observed under a polarizing optical microscope. Next, to differentiate the type of amyloidosis, an immunohistochemical test using monoclonal antibodies for the specific case of light chains is performed.^4,6–10^ The presence of monoclonal light chains in urine and serum is useful, but not always enough to diagnose systemic disorders, like AL. For all patients, electrophoresis with serum (71% sensitivity) and urine (84% sensitivity) immunofixation needs to be performed, in order to attempt to reveal monoclonal light chains.^11^ In cases where the results are initially negative, it may be necessary to redo the immunofixation.

Normally, all patients should undergo the serum-free light chain assay to assess disease evolution. Renal involvement is diagnosed through the evidence of renal amyloid deposits detected through biopsy, together with laboratory evidence of kidney dysfunction (24 hour proteinuria more than 0.5 g per day; mainly albumin). Heart involvement is identified through biopsy, in the presence of clinical evidence and changes seen on echocardiogram (like ventricular walls thicker than 12 mm in the absence of hypertension or other potential causes of such thickening). Reduction of the ejection fraction occurs as a late event.^9–14^

Thirty years ago, the treatment for this entity was mainly palliative. New therapies directed towards stabilization of amyloid fibrils have substantially improved these patients’ quality of life. In patients with adequate criteria, stem cell transplantation has shown encouraging results in several recent studies, and the five-year survival rate has been estimated to be approximately 60%.^15,16^ A systematic search in major databases (Table 2) found some papers that showed the difficulties in clinical approaches and the therapeutic implications of improvements in clinical outcomes.^17,18^ Other studies have revealed that despite the new therapeutic and clinical approaches, the survival rate remains unsatisfactory.^19,20^ The patient in the present report received a regimen of high doses of steroids (dexamethasone, 40 mg) in association with a proteosome inhibitor (bortezomib, 0.7 mg/m^2^), and despite its toxicity, it led the patient to a good response with good tolerance, and an increase in the patient's quality of life. Because he presented stage V renal disease with hemodialysis support, age above 65 years and slightly increased basal brain natriuretic peptide, the patient did not fulfill the criteria for stem cell transplantation and was classified as high risk. Nonetheless, he benefited from the chemotherapy. Currently, the patient's quality of life is better and the disease has not progressed.

**Table 2:** Results from systematic search using description of the main clinical features observed in the patient of the present report, among papers indexed in PubMed, Lilacs (Literatura Latino-Americana e do Caribe em Ciências da Saúde), Embase, Scopus and Cochrane Library, from 1982 to 2010

Data	Search strategy	Results	
PubMed	Amyloidosis (MeSH) and kidney failure (MeSH) and nephrotic syndrome (MeSH)	57 manuscripts	8 reviews
			27 case reports
			1 comment/editorial
			3 letters
			4 comparative study

Lilacs	Amyloidosis (DeCS) and kidney failure (DeCS) and nephrotic syndrome (DeCS)	1 case report	
Embase	“Amyloidosis” and “kidney failure” and “nephrotic syndrome”	161 manuscripts	6 reviews
			11 case reports
			3 letters
Scopus	Amyloidosis (key words) and kidney failure (key words) and nephrotic syndrome (key words)	175 manuscripts	25 reviews
			13 letters
			1 note
			4 short surveys
			4 conference papers
Cochrane	Amyloidosis	1 review	

MeSH = Medical Subject Headings; DeCS = Descritores em Ciências da Saúde.

## CONCLUSION

Amyloidosis is a disease with serious diagnostic difficulties because it commonly has unspecific forms of presentation. In fact, many cases are underdiagnosed and this diagnosis is only considered when the patient presents organ failure. Therefore, this case alerts general practitioners to give more attention to this clinical entity and to the importance of early diagnosis, particularly in patients with decompensated renal failure and severe clinical features of unknown cause that are difficult to control. This case consisted of primary amyloidosis, which is a rare entity, and showed involvement of the kidneys as an early presentation of the disease, with diagnostic difficulties. Early diagnosis and treatment are helpful in stabilizing the disease, thereby improving the care and the outcome.
